# Prediction scores of postoperative liver metastasis and long‐term survival of pancreatic head cancer based on the distance between the mesenteric vessels and tumor, preoperative serum carbohydrate antigen 19‐9 level, and lymph node metastasis rate

**DOI:** 10.1002/cam4.4957

**Published:** 2022-07-13

**Authors:** Dan Jiang, Xiaona Fan, Pengfei Li, Yang Zhou, Kaige Chen, Hengzhen Li, Jinshuang Liu, Wenjing Zhang, Yisheng Dai, Ning Sun, Zhiwei Li

**Affiliations:** ^1^ Department of Gastrointestinal Oncology Harbin Medical University Cancer Hospital Harbin Heilongjiang P.R. China

**Keywords:** CA 19‐9, distance of the superior mesenteric artery, distance of the superior mesenteric vein, long‐term survival, lymph node ratio, pancreatic head carcinoma, postoperative liver metastasis

## Abstract

**Background:**

The shortest distance between the superior mesenteric artery (SMA) or superior mesenteric vein (SMV) and the tumor margin was combined with preoperative serum carbohydrate antigen (CA) 19‐9 and lymph node ratio (LNR) to evaluate joint effects on long‐term survival and liver metastasis in patients with pancreatic head cancer after radical surgery.

**Methods:**

This retrospective study included 149 patients who underwent pancreaticoduodenectomy for pancreatic head cancer at Harbin Medical University Tumor Hospital from May 2011 to March 2021. The preoperative serum CA 19‐9 level and LNR were combined with the SMA or SMV distance. The joint association between long‐term survival and postoperative liver metastasis was evaluated.

**Results:**

Based on the receiver operating characteristic curve of postoperative liver metastasis or long‐term survival, the optimal cut‐off values of SMV distance were 3.1 and 0.7 mm, respectively, whereas the optimal cut‐off value of SMA distance was 10.25 mm. The univariate model identified the liver metastasis score (*p* < 0.001) as a negative factor for postoperative liver metastasis of pancreatic head carcinoma. The SMV distance (*p* = 0.003), SMA distance (*p* < 0.001), LNR score (*p* < 0.001), and survival score (*p* < 0.001) were negatively correlated with long‐term survival after pancreatic head cancer. The multivariate model highlighted SMA distance (*p* < 0.001), survival score (*p* = 0.001), and LNR score (*p* < 0.001) as independent risk factors for long‐term survival in pancreatic head cancer.

**Conclusion:**

Liver metastasis score may be an independent predictor of postoperative liver metastasis in patients with pancreatic head cancer. Survival and LNR scores may be independent predictors of long‐term postoperative survival in patients with pancreatic head cancer. However, the LNR score appears to improve long‐term survival.

## Graphical abstract

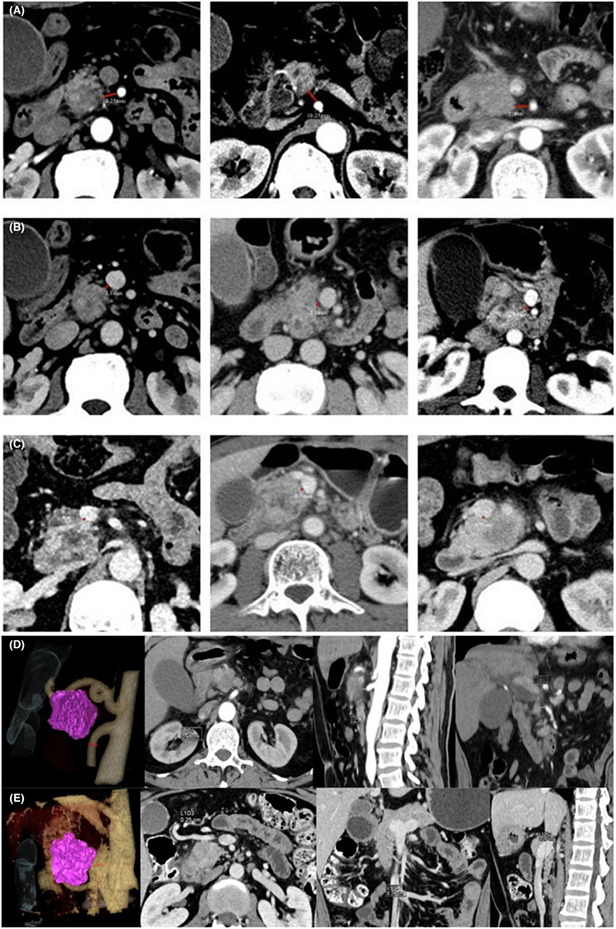

## 1 INTRODUCTION

According to Global Cancer Statistics,^1^ pancreatic cancer is the seventh most common cause of malignancy‐related deaths worldwide. Pancreatic head carcinoma accounts for 80%–90% of all pancreatic malignancies. Recently, radical pancreaticoduodenectomy remains the most effective treatment for patients with resectable pancreatic head cancer.^2^ The major goal of surgery is to achieve R0 resection with potential therapeutic effect.^3^ However, due to the lack of susceptible and specific screening methods for early detection, only 10%–25% of patients are able to undergo radical resection.^4^, ^5^, ^6^ However, most patients inevitably experience disease recurrence or distant metastasis, resulting in a 5‐year survival rate of only 18%–27%.^4^, ^7^ The liver is the most common organ (approximately 70%) with distant metastases in advanced pancreatic cancer. Compared with lung and bone metastases, pancreatic cancer with liver metastasis has the worst prognosis, with an overall survival (OS) period of <6 months.^8^ Hence, the preoperative prediction of liver metastasis and long‐term survival in patients with resectable pancreatic head cancer is of great significance. On the one hand, the screening range of patients undergoing surgery can be narrowed, which not only improves the efficiency of surgery but also avoids the risk of patients receiving inappropriate treatment. On the other hand, it can also guide patients' preoperative and postoperative treatment, prolonging their life and reducing their pain.

In this study, we aimed to identify a new parameter for predicting postoperative liver metastasis and long‐term survival in patients with pancreatic head cancer using preoperative variables.

The pancreas has an abundant blood supply. For example, the pancreatic head mainly receives blood from the celiac and superior mesenteric arteries. In addition, the anatomical position of the pancreatic head is exceptional. The pancreatic head is closely related to major abdominal vessels, including the superior mesenteric artery (SMA) and vein, portal vein, and celiac artery. Therefore, pancreatic head cancer can easily invade these vessels. When pancreatic head cancer invades adjacent major vascular structures, such as the SMA, complications occur and radical surgery cannot be performed. This explains why early‐stage pancreatic cancer is at risk of metastasis, leading to a poor prognosis. Lymph node involvement has been a crucial factor in the poor prognosis of pancreatic cancer.^9^, ^10^ The predictive value of lymph node metastasis rate has been proposed for patients undergoing pancreatectomy.^11^, ^12^ For example, when exploring the association between the lymph node metastasis rate and long‐term survival after radical surgery for pancreatic cancer and postoperative adjuvant chemotherapy, the lymph node metastasis rate correlated with reduced OS in patients undergoing radical surgery and chemotherapy.^13^ The superior mesenteric vein (SMV), as the main venous return route of the pancreas, collects venous blood from the pancreatic head and neck and eventually merges with the inferior mesenteric and splenic veins to form a portal vein that flows into the liver. Therefore, the molecular mechanism of hepatic metastasis in pancreatic cancer is related to the unique pancreatic venous return.^7^ The distance between the SMA and the tumor correlates with postoperative local recurrence in patients with pancreatic cancer.^14^ In this study, the preoperative distance between the SMA and the tumor was associated with the preoperative serum carbohydrate antigen (CA) 19‐9 value. A new score was established to evaluate the potential association between the new score and postoperative local recurrence of pancreatic cancer. This score may be an independent predictor of postoperative local recurrence in patients with pancreatic cancer. Based on predecessors, the shortest distance between the SMA or SMV and the tumor margin was combined with preoperative serum CA 19‐9 level and lymph node ratio (LNR) to evaluate the joint effects on long‐term survival and liver metastasis in patients with pancreatic head cancer after radical surgery to provide individualized treatment for these patients.

## 2 PATIENTS AND METHODS

### 2.1 Patients

**FIGURE 1:**
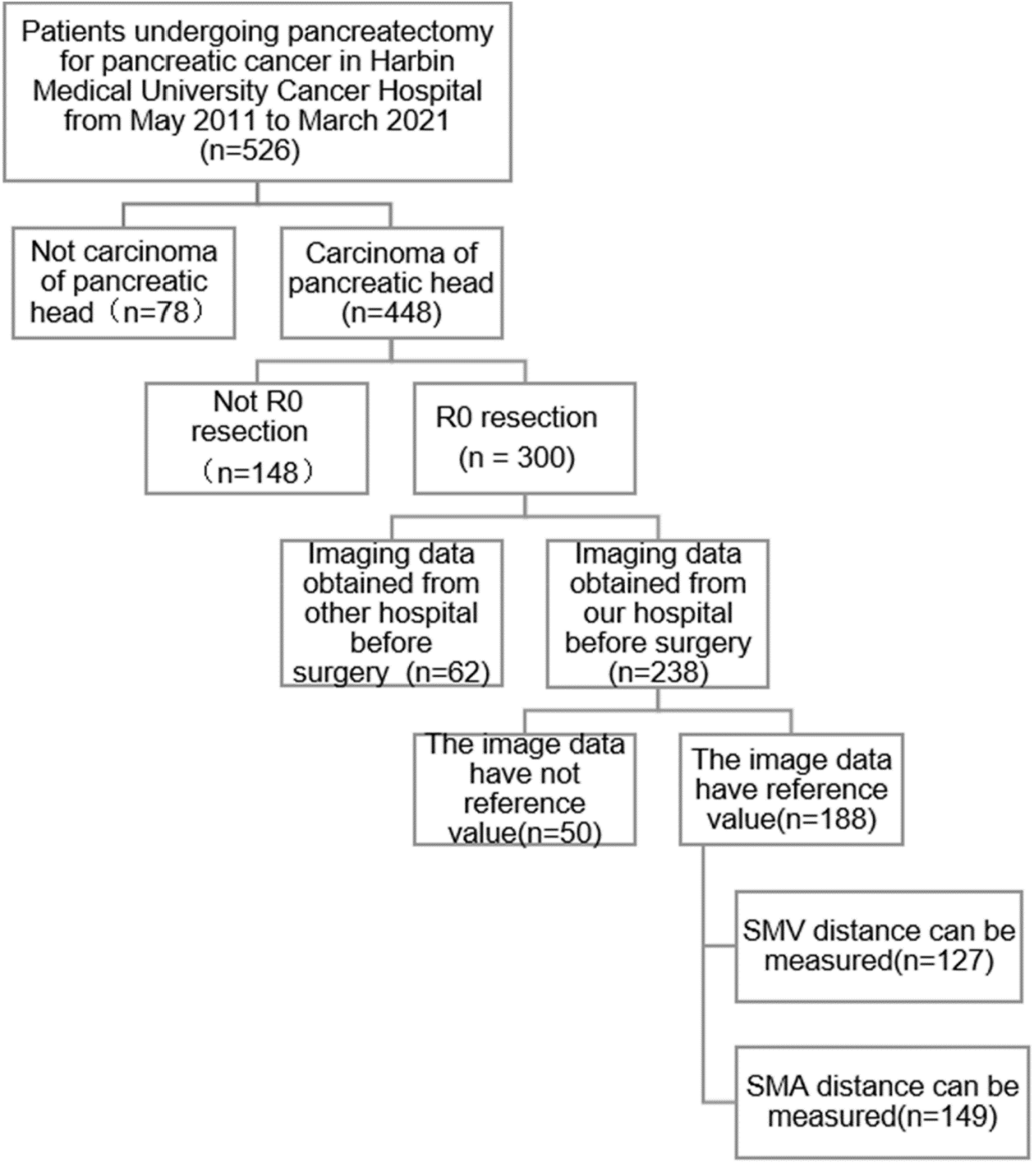
Flowchart of case screening. SMA, superior mesenteric artery; SMV superior mesenteric vein.

This hospital‐based retrospective study included 149 patients who underwent pancreaticoduodenectomy for pancreatic head cancer at the Harbin Medical University Tumor Hospital from May 2011 to March 2021. The inclusion criteria were as follows: (1) Pancreaticoduodenectomy plus regional lymph node dissection for pancreatic head cancer performed at our hospital, (2) preoperative imaging data available for reference, (3) postoperative pathology confirming ductal adenocarcinoma of the pancreatic head, and (4) absence of neoadjuvant therapy. The exclusion criteria were as follows: (1) postoperative pathology confirming metastatic pancreatic cancer, ampullary carcinoma, cholangiocarcinoma, duodenal tumor, and pancreatic endocrine tumor; (2) palliative surgery only; (3) vascular invasion confirmed by preoperative examination, intraoperative exploration, or postoperative pathology; and (4) lost to follow‐up. A flowchart of case screening is shown in Figure 1.

### 2.2 Follow‐up visit

Patients were followed up by outpatient visits, telephone calls, and in‐patient examinations. Follow‐up visits were performed every 3 months in the first year after surgery. Follow‐up visits were performed every 3–6 months at 2–3 years postoperatively. Follow‐up visits were performed every 6 months for 3–5 years postoperatively. Follow‐up included physical and routine blood examinations, blood biochemistry, carcinoembryonic antigen (CEA), and CA 19‐9 level assessment, imaging examination, postoperative survival status evaluation, and liver metastasis assessment. OS was defined as the date from radical surgery to death or the last follow‐up. Disease‐free survival (DFS) was defined as the time from the date of radical surgery to tumor recurrence, progression, or death. All patients were followed up until March 31, 2021.

### 2.3 Statistical analyses

The Statistical Package for the Social Sciences version 25.0 was used for the statistical analyses. The chi‐squared tests were used to compare the differences between the two groups in counting data, whereas the rank‐sum tests were used to compare the two groups of grading data. The Kaplan–Meier (K–M) curve was used to estimate survival time, whereas the log‐rank test was applied to compare the median survival time. Univariate and multivariate logistic regression models were used to correlate different factors with postoperative liver metastasis. Univariate and multivariate Cox proportional risk models were used to correlate different factors with long‐term postoperative survival. Univariate analysis of clinicopathological variables and new scores was integrated into a multiple logistic regression model. Statistical significance was set at *p* < 0.05.

## 3 METHODS

First, the patients were grouped according to blood vessels, observations, and combined factors. The shortest distance between the edge of the SMA or SMV and the edge of the most extensive section of the tumor was obtained according to preoperative imaging data of the reference value. The SMV or SMA distance was measured independently by two radiologists who were blinded of the patients' clinical information, and a final decision was made by the two radiologists in the case of disagreement. All results were recorded by the hepatobiliary, pancreatic, and splenic surgeons who performed the surgeries. The best cut‐off value for the distance was calculated using the receiver operating characteristic (ROC) curve. The survival status and liver metastasis of pancreatic head cancer after surgery were compared to evaluate their discrimination ability. Subsequently, the optimal cut‐off value of the distance was combined with the cut‐off value of preoperative serum CA 19‐9 and LNR and categorized to establish a new score. The influence of this new score on postoperative liver metastasis and long‐term survival was also evaluated.

Next, the association between clinicopathological variables and new self‐developed scores was explored using univariate analysis. Finally, to determine the predictors of postoperative liver metastasis and long‐term survival of pancreatic head cancer, univariate analysis was performed on age, sex, health status, liver status, laboratory indicators, tumor length, preoperative serum CEA level, preoperative serum CA 19‐9 level, lymph node metastasis, LNR, tumor stage, tumor differentiation, nerve invasion, postoperative liver metastasis, postoperative distant metastasis, postoperative chemotherapy, SMA distance, SMV distance, LNR score, liver metastasis score, and survival score. Statistically significant factors were selected for multivariate analysis.

## 4 RESULTS

### 4.1 Patient characteristics and groups

A total of 149 (90 men and 59 women; age, 31–78 [median, 58] years) patients were recruited for this study. The median maximum tumor diameter was 3 cm. All patients with pancreatic head carcinoma with a pathological type of ductal adenocarcinoma underwent pancreaticoduodenectomy plus regional lymph node dissection. Thirty‐eight patients received gemcitabine‐based chemotherapy postoperatively. Ninety‐eight patients had jaundice, 70 of whom underwent percutaneous transhepatic cholangial drainage before surgery. Patient characteristics are summarized in Table 1. The SMA distance could be measured in the SMA group, with 149 patients in total, and the SMV distance could be measured in the SMV group, with 127 patients in total.

**TABLE 1:** Patient characteristics

Characteristic	Number
Sex	
Male	90
Female	59
Age (year)	
≤60	87
>60	62
BMI (kg/m^2^)	
≤20	26
20–25	79
≥25	48
Underlying disease	
+	65
–	84
Liver disease	
+	64
–	85
Child–Pugh stage	
A	124
B	25
Tumor length to diameter	
≤3 cm	82
>3 cm	67
Degree of tumor differentiation	
High differentiation	19
High‐medium differentiation	27
Medium differentiation	60
Medium‐low differentiation	36
Low differentiation	7
TNM stage	
I	57
II	85
III	7
Nerve invasion	
+	46
–	103
Postoperative distant metastasis	
+	63
‐	86
Postoperative liver metastases	
+	35
–	114
Lymphatic metastasis	
+	45
–	104
Preoperative imaging	
Epigastric enhanced CT	64
PET‐CT	18
Epigastric MRI enhancement	43
Epigastric MRI	6
Pancreatic enhancement CT	17
Three‐dimensional imaging of the pancreas	1
SMV distance	
≤3.1 mm	74
>3.1 mm	53
≤0.7	65
>0.7	62
SMA distance	
≤10.25	125
>10.25	24
Preoperative serum CA 19‐9 level	
≤200 U/ml	80
>200 U/ml	61
LNR	
≤0.2	124
>0/2	25
Postoperative chemotherapy	
+	38
–	111

Abbreviations: BMI, body mass index; LNR, lymph node ratio; SMA, superior mesenteric artery; SMV, superior mesenteric vein; CA 19‐9, carbohydrate antigen 19‐9.

The median OS and DFS periods were 15.6 and 9.65 months, respectively. The 5‐year survival rates with LNR scores of 0, 1, and 2 were 38%, 13%, and 6%, respectively. The 5‐year survival rates with survival scores of 0, 1, and 2 were 16%, 3%, and 0%, respectively.

### 4.2 Different scores associated with clinical outcomes and the establishment of scores

**FIGURE 2:**
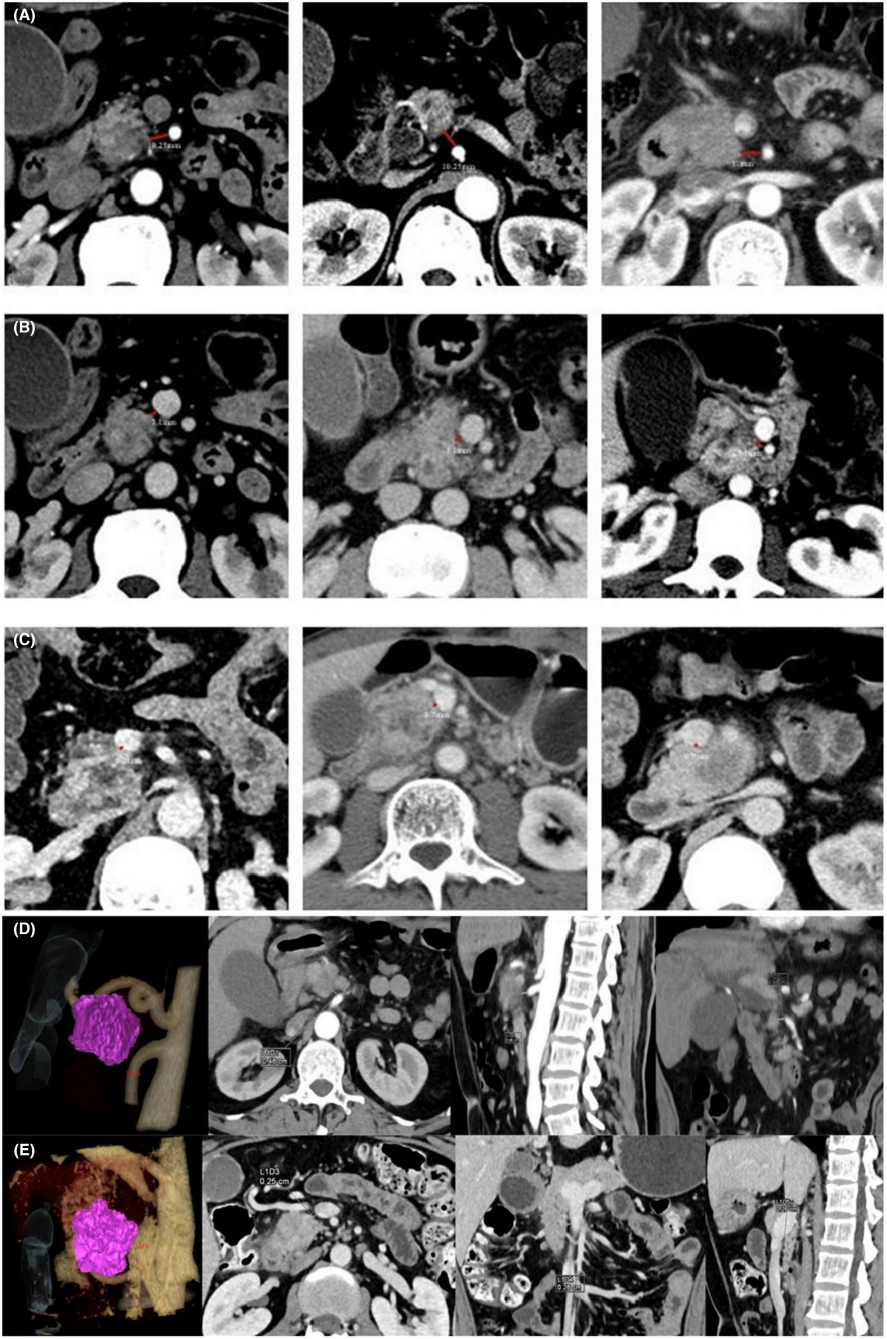
The shortest distance between the superior mesenteric vein (SMV), superior mesenteric artery (SMA), and the edge of the tumor. (A) SMA distance = 10.25 mm, (B) SMV distance = 3.1 mm, (C) SMV distance = 0.7 mm, (D) SMA distance = 0.46 cm, and (E) SMV distance = 0.25 cm. SMA superior mesenteric artery; SMV superior mesenteric vein.

**FIGURE 3:**
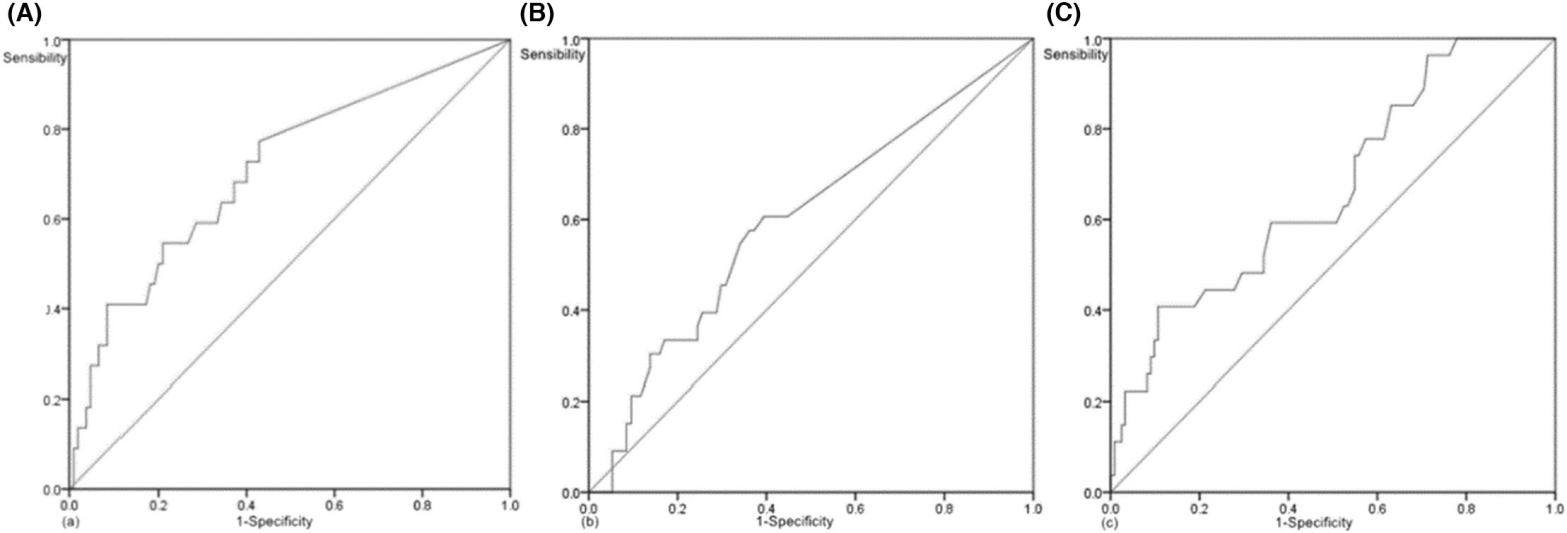
(A) Receiver operating characteristic (ROC) curve of postoperative superior mesenteric vein (SMV) distance and long‐term survival in pancreatic head carcinoma (area under the curve [AUC] = 0.710, *p* = 0.002). (B) ROC curve of postoperative SMV distance and liver metastasis of pancreatic head carcinoma (AUC = 0.598, *p* = 0.095). (C) ROC curve of postoperative superior mesenteric artery (SMA) distance and long‐term survival in pancreatic head carcinoma (AUC = 0.668, *p* = 0.007). SMA superior mesenteric artery; SMV superior mesenteric vein.

The shortest distance between the SMA and the edge of the tumor was measured based on preoperative imaging data. The specific requirements were as follows. The image displaying the largest tumor with the SMA located at the same level as the tumor was selected to measure the shortest distance between the edge of the SMA and the edge of the tumor, that is, the SMA distance. The SMV distance was obtained in a similar manner. The axial position, sagittal and coronal SMA distances, and SMV distance were measured; however, the axial position was set as the final standard (Figure 2). Based on the ROC curves for long‐term survival, the optimal cut‐off value of SMA distance was 10.25 mm (area under the curve [AUC] = 0.668, *p* = 0.007). The optimal cut‐off value of the SMV distance was 0.7 mm (AUC = 0.710, *p* = 0.002). Based on the ROC curves for liver metastasis, the optimal cut‐off value of SMV distance was 3.1 mm (AUC = 0.598, *p* = 0.095, Figure 3). In this study, the preoperative cut‐off value of serum CA 19‐9 was 200 U/ml, and the cut‐off value of LNR was 0.2. The optimal cut‐off value of the distance was combined with the cut‐off value of preoperative serum CA 19‐9 or LNR to establish a new score (Tables 2, 3, 4).

**TABLE 2:** Survival score

Index		
Survival score	SMV distance (mm)	Preoperative serum CA 19‐9 level (U/ml)
0	≥0.7	≤200
1	<0.7	≤200
1	≥0.7	>200
2	<0.7	>200

Abbreviations: CA 19‐9, carbohydrate antigen 19‐9; SMV, superior mesenteric vein.

**TABLE 3:** Hepatic metastasis score

Index		
Hepatic metastasis score	SMV distance (mm)	Preoperative serum CA 19‐9 level (U/ml)
0	≥3.1	≤200
1	<3.1	≤200
1	≥3.1	>200
2	<3.1	>200

Abbreviations: CA 19‐9, carbohydrate antigen 19‐9; SMV, superior mesenteric vein.

**TABLE 4:** LNR score

Index		
LNR score	SMA distance (mm)	LNR
0	≥10.25	≤0.2
1	<10.25	≤0.2
1	≥10.25	>0.2
2	<10.25	>0.2

Abbreviations: LNR, lymph node ratio; SMA, superior mesenteric artery.

### 4.3 Potential effects of new scores on postoperative liver metastasis and long‐term survival

**FIGURE 4:**
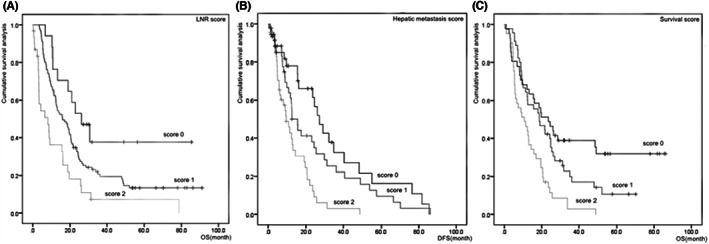
(A) Lymph node ratio score with postoperative long‐term survival of the Kaplan–Meier curve (*p* < 0.001). (B) Hepatic metastasis score and postoperative liver metastasis of the Kaplan–Meier curve (*p* < 0.001). (C) Survival score with postoperative long‐term survival of the Kaplan–Meier curve (*p* < 0.001).

The predictive effects of the new scores on postoperative liver metastasis and long‐term survival were explored using K–M curves. The association between the liver metastasis score and postoperative liver metastasis of pancreatic head cancer was as follows: Group 0 had the highest cumulative survival rate, Group 1 had the intermediate cumulative survival rate, and Group 2 had the lowest cumulative survival rate. The association between the LNR and survival scores and long‐term survival for pancreatic head cancer was as follows: Group 0 had the highest cumulative survival, Group 1 had the intermediate cumulative survival rate, Group 2 had the lowest cumulative survival, and there was comparability among all groups. There was no significant correlation between tumor stage and the LNR or survival score (*p* > 0.05) (Figure 4).

### 4.4 Univariate analysis of characteristics and new scores in patients with pancreatic head carcinoma

The association between new scores and clinicopathological factors was analyzed. Age (*p* = 0.005), presence of underlying diseases (*p* = 0.012), international normalized ratio (*p* = 0.010), prothrombin time (*p* = 0.017), lactate dehydrogenase level (*p* = 0.023), and activated partial thromboplastin time (*p* = 0.044) correlated with liver metastasis score. Age (*p* = 0.037) and presence of underlying diseases (*p* = 0.004) were correlated with survival score. There was no association between these factors and LNR score (Tables 5 and 6, Tables A1 and A2).

**TABLE 5:** Univariate analysis of characteristics and hepatic metastasis score of patients with pancreatic head cancer

Factor	Hepatic metastasis score			*p*‐value
	0 (*n* = 36)	1 (*n* = 46)	2 (*n* = 45)	
Age (year)	59^a^	58.5^a^	59^a^	0.005
Sex (male/female)	23/13	28/18	27/18	0.934
BMI	9/16/11	8/25/13	5/28/12	0.798
Underlying disease (+/−)	11/25	16/30	27/18	0.012
Liver disease (+/−)	13/23	17/29	26/19	0.071
Child–Pugh stage (A/B)	28/8	34/12	40/5	0.161
Tumor length to diameter (≤3/>3 cm)	23/13	25/21	27/18	0.660
CEA	3.235^a^	3.205^a^	3.21^a^	0.053
Neoplasm staging (I/II/III)	13/21/2	19/24/3	20/23/2	0.743
Degree of tumor differentiation				0.520
Nerve invasion (+/−)	13/23	14/32	11/34	0.658
Lymphatic metastasis (+/−)	10/26	11/35	18/27	0.091
LNR (≤0.2/>0.2)	28/8	43/3	37/8	0.170

Abbreviations: BMI, body mass index; CEA, carcinoembryonic antigen; LNR, lymph node ratio.

^a^ Median.

**TABLE 6:** Univariate analysis of characteristics and survival score of patients with pancreatic head cancer

Factor	Survival score			*p* value
	0 (*n* = 41)	1 (*n* = 45)	2 (*n* = 41)	
Age (year)	58.5^a^	57.5^a^	59^a^	0.037
Sex (male/female)	26/15	27/18	25/16	0.946
BMI	10/19/12	7/25/13	5/25/11	0.770
Underlying disease (+/−)	13/28	15/30	26/15	0.004
Liver disease (+/−)	14/27	18/27	24/17	0.066
Child–Pugh stage (A/B)	33/8	33/12	36/5	0.233
Tumor length to diameter (≤3/>3 cm)	27/14	25/20	23/18	0.640
CEA	3.235^a^	3.205^a^	3.21^a^	80.064
Neoplasm staging (I/II/III)	16/23/2	19/22/4	17/23/1	0.824
Degree of tumor differentiation				0.581
Nerve invasion (+/−)	14/27	13/32	11/30	0.846
Lymphatic metastasis (+/−)	11/30	12/33	16/25	0.161
Postoperative hepatic metastases (+/−)	13/28	12/33	8/33	0.443
Postoperative distant metastases (+/−)	18/23	26/19	14/27	0.084
LNR (≤0.2/>0.2)	32/9	41/4	35/6	0.391
PLR	143.5^a^	146.6^a^	145.3^a^	0.066
NLR	2.68^a^	2.77^a^	2.77^a^	0.062
HGB	124.5^a^	123.6^a^	120	0.088

Abbreviations: BMI, body mass index; CEA, carcinoembryonic antigen; HGB, hemoglobin; LNR, lymph node ratio; NLR, peripheral blood neutrophil‐to‐lymphocyte ratio; PLR, preoperative platelet‐to‐lymphocyte ratio.

^a^ Median.

### 4.5 Univariate and multivariate analyses of clinicopathological variables associated with liver metastasis after the resection of pancreatic head carcinoma

The effect of different variables on postoperative liver metastasis of pancreatic head cancer was assessed by univariate analysis, and the liver metastasis score was an independent risk factor for liver metastasis after pancreatic head cancer (*p* < 0.001). Thus, the combination of SMV distance and preoperative serum CA 19‐9 level was superior to either SMV distance or preoperative serum CA 19‐9 level alone in evaluating postoperative liver metastasis (Table 7, Table A3).

**TABLE 7:** Univariate and multivariate analyses of postoperative liver metastasis

Factor	Univariate			Multivariate		
	95% CI	OR	*p*‐value	95% CI	OR	*p*‐value
Age (year)	0.929–1.016	0.971	0.203			
Sex (male/female)	0.326–1.724	0.750	0.498			
BMI	0.724–2.233	1.272	0.402			
Underlying disease	0.282–1.357	0.618	0.231			
Liver disease	0.839–3.837	1.794	0.132			
Tumor length to diameter (cm)	0.735–1.191	0.936	0.590			
Child–Pugh stage	0.256–1.640	0.648	0.360			
Preoperative serum CEA level (ng/ml)	0.969–1.046	1.007	0.725			
Preoperative serum CA 19–9 level (U/ml)	0.603–2.907	1.324	0.484			
Lymphatic metastasis (+/−)	0.749–4.312	1.797	0.189			
Degree of tumor differentiation	0.760–1.411	1.035	0.826			
Nerve invasion (+/−)	0.311–1.706	0.729	0.466			
SMV distance (mm)	0.967–1.163	1.061	0.209			
Hepatic metastasis score	1.327–2.348	1.765	0.000			
TNM stage	0.483–1.820	0.938	0.849			
LNR	0.038–5.430	0.454	0.533			

Abbreviations: BMI, body mass index; CA 19‐9, carbohydrate antigen 19‐9; CEA, carcinoembryonic antigen; LNR, lymph node ratio; SMV, superior mesenteric vein.

### 4.6 Univariate and multivariate analyses of clinicopathological variables associated with long‐term survival after the resection of pancreatic head carcinoma

The potential effects of different variables on the long‐term survival of pancreatic head cancer were assessed by univariate analysis, and postoperative liver metastasis (*p* = 0.039), postoperative adjuvant chemotherapy (*p* = 0.031), SMV distance (*p* = 0.003), SMA distance (*p* < 0.001), LNR score (*p* < 0.001), and survival score (*p* < 0.001) were negatively associated with the long‐term survival of pancreatic head cancer. The multivariate model identified SMA distance (*p* < 0.001), survival score (*p* < 0.001), LNR score (*p* < 0.001), postoperative liver metastasis (*p* < 0.001), and postoperative adjuvant chemotherapy (*p* = 0.017) as independent risk factors for long‐term survival of pancreatic head cancer. These results suggest that the LNR and survival scores might be independent predictors of long‐term postoperative survival in patients with pancreatic head cancer (Table 8).

**TABLE 8:** Univariate multivariate analysis of long‐term survival

Factor	Univariate			Multivariate		
	95% CI	HR	*p*‐value	95% CI	HR	*p*‐value
Age (year)	0.976–1.017	0.997	0.745			
Gender (male/female)	0.547–1.196	0.809	0.288			
BMI	0.806–1.362	1.048	0.727			
Underlying disease	0.711–1.443	1.013	0.942			
Liver disease	0.870–1.764	1.239	0.235			
Tumor length to diameter (cm)	0.946–1.162	1.048	0.368			
Child‐Pugh stage	0.635–1.569	0.998	0.994			
Preoperative serum CEA (ng/ml)	0.995–1.031	1.013	0.156			
Preoperative serum CA199 (U/ml)	0.488–1.003	0.700	0.052			
Lymphatic metastasis (+/−)	0.784–1.663	1.142	0.489			
Degree of tumor differentiation	0.894–1.194	1.033	0.657			
Nerve invasion (+/−)	0.455–0.990	0.671	0.044	0.432–1.069	0.680	0.095
SMA distance (mm)	0.869–0.959	0.913	0.000	0.764–0.913	0.835	0.000
SMV distance (mm)	0.876–0.973	0.923	0.003	0.962–1.105	1.031	0.391
Postoperative distant metastases	0.875–1.762	1.242	0.225			
Postoperative liver metastasis (+/−)	1.023–2.299	1.533	0.039	1.561–4.221	2.567	0.000
TNM stage	0.984–1.874	1.358	0.062			
LNR	0.231–2.415	0.748	0.627			
Survival score	1.325–2.223	1.716	0.000	1.442–3.452	2.231	0.000
LNR score	1.415–2.787	1.986	0.000	3.136–10.767	5.811	0.000
Postoperative chemotherapy (+/−)	0.403–0.958	0.622	0.031	0.316–0.893	0.531	0.017
PLR	0.996–1.000	0.998	0.096			
NLR	0.932–1.010	0.970	0.138			
HGB	0.997–1.018	1.008	0.143			

Abbreviations: BMI, body mass index; CEA, carcinoembryonic antigen; HGB, hemoglobin; LNR, lymph node ratio; NLR, peripheral blood neutrophil‐to‐lymphocyte ratio; PLR, preoperative platelet‐to‐lymphocyte ratio; SMA, superior mesenteric artery; SMV, superior mesenteric vein.

## 5 DISCUSSION

Currently, vascular invasion is correlated with long‐term survival after the resection of pancreatic cancer. For example, splenic vein involvement is a predictor of poor postoperative prognosis and early liver metastasis in patients with pancreatic body–tail cancer.^15^ This view was supported by a meta‐analysis of six studies. Splenic artery or splenic vein infiltration after distal pancreatectomy is associated with lower survival in resectable caudal ductal adenocarcinoma of the pancreatic body.^16^ Moreover, the SMV is related to the prognosis of pancreatic tumors. Preoperative tumor and portal vein/SMV contact are independently associated with poor survival.^17^ Furthermore, the distance between the tumor and portal vein/SMV might predict the survival rate of patients with pancreatic head cancer and the response to neoadjuvant therapy to improve prognosis.^18^ However, the association between vascular distance and long‐term survival after pancreatic cancer resection has not been explored. SMA, an important blood vessel, was not considered an influential factor. In a recent study, the preoperative distance between the SMA and tumor as an influential factor of postoperative local recurrence of pancreatic cancer was based on the histological stage.^14^ However, the above study was designed to be relatively simple and did not further explore the correlation between vascular distance and long‐term survival. The SMV is a principal venous reflux vessel in the pancreatic head and is closely related to hepatic metastasis of pancreatic cancer. However, the association between the SMV and liver metastasis after pancreatic head cancer has yet to be demonstrated. In addition, the predictive value of the distance between the preoperative SMV and pancreatic head tumor for postoperative liver metastasis has not been reported.

Our study has proposed that the preoperative distance between the tumor and blood vessels affects postoperative long‐term survival. In this study, CA 19‐9, a well‐known prognostic factor for pancreatic cancer, was included. Although there is no evidence that preoperative serum CA 19‐9 level is related to postoperative liver metastasis in patients with resectable pancreatic head cancer, preoperative serum CA 19‐9 level is an independent risk factor for early postoperative recurrence and lymph node metastasis of pancreatic cancer.^19^, ^20^ CA 19‐9 level is also associated with later tumor stage, heavier tumor burden, and shorter tumor progression time.^19^, ^21^ Interestingly, in clinical practice, we have also observed that preoperative serum CA 19‐9 level seems to be higher in patients with postoperative liver metastasis of pancreatic head cancer than in those without postoperative liver metastasis. The authors believe that this is closely related to the fact that CA 19‐9 can accelerate pancreatic cancer progression by glycosylating proteins, binding to E‐selectin, strengthening angiogenesis, and mediating immunological response.^21^ Hematogenous metastasis is the main pathway of liver metastasis in pancreatic cancer. A high preoperative serum CA 19‐9 level may indicate the presence of small liver metastasis in vivo, which seems to explain the correlation between preoperative serum CA 19‐9 level and postoperative liver metastasis. The cut‐off value of CA 19‐9 was determined as 200 U/ml based on previous experience.^14^, ^19^ Lymph node metastasis rate was included, as previously reported.^11^, ^12^, ^13^ Our study has validated that the lymph node metastasis rate plays a role in predicting the prognosis of pancreatic cancer. The investigators concluded that the rate of lymph node metastasis had a greater predictive value than the status of lymph node metastasis in patients undergoing pancreatectomy but also determined the optimal cut‐off value for the rate of lymph node metastasis. The results of He et al.’s study indicated that this value should be 0.25,^22^ but the rate of lymph node metastasis in their study was affected by the total number of lymph nodes detected, which may have led to a slight bias in the results. Zhan et al. avoided this point and set the intercept value to 0.2.^12^ Although the two values deviate, they do not have a significant effect on our study. The factors included in these two studies were relatively single; therefore, it was impossible to avoid errors caused by single factors. In summary, we determined that the intercept value of the LNR was 0.2.

It is worth noting that the two scores in our study, combining different factors, may be independent predictors of long‐term postoperative survival in patients with pancreatic head cancer, which is unprecedented compared to previous studies and provides new ideas and directions for both researchers and clinicians. From the perspective of image quality, we believe that the LNR score is superior. Since the distance of the SMV is only 0.7 mm in the survival score, different imaging data may affect the measurement of distance to a certain extent.

To the best of our knowledge, this is the first study to explore preoperative variables to identify the predictors of postoperative liver metastasis in patients with pancreatic head cancer. Although some researchers have studied preoperative contact between the tumor and SMV and the long‐term postoperative survival of patients with resectable pancreatic head cancer,^17^ and even proposed a new concept of contact length,^18^ an optimal cut‐off value was not introduced, and other influencing factors were included. There was no reasonable explanation for the association between the distance between the preoperative SMV and the pancreatic head tumor and postoperative liver metastasis. The present study combined two variables that were readily available preoperatively to avoid the inevitable errors caused by a single factor. We stratified the risk of postoperative liver metastasis in patients with pancreatic head cancer by establishing a new score that may be helpful in determining the degree of tumor invasiveness before surgery. The SMA distance may not affect postoperative liver metastasis of pancreatic cancer. The SMV distance is correlated with postoperative liver metastasis and long‐term survival of pancreatic cancer. The SMV is superior to the SMA in predicting long‐term survival and liver metastasis after pancreatic cancer surgery. Although there was no significant correlation between the survival and LNR score and TNM stage, the predictive effects of these scores and TNM stage have not been determined, and further studies are required.

The current study has some limitations. This was a retrospective hospital‐based study. However, the sample size of this study was relatively small. Moreover, this was a single‐institution study. Preoperative imaging data of the reference values were not uniform. To test our hypothesis, we established new scores using SMA distance, SMV distance, preoperative serum CA 19‐9 level, and LNR. We confirmed the association among new scores, postoperative liver metastasis, and long‐term survival. The new score is associated with SMA distance, CA 19‐9 level, and LNR with long‐term survival after pancreatic cancer surgery, providing a diversified reference for subsequent studies. Furthermore, the introduction of SMV distance has verified that SMV is closely related to pancreatic cancer liver metastasis and has elaborated that the preoperative distance between the SMV and pancreatic head tumor affects postoperative liver metastasis. Therefore, the inclusion of CA 19‐9 reduces the inevitable error caused by a single factor, making the results more convincing. Risk stratification before surgery using this new score is feasible because preoperative imaging and tumor markers are considered the standard of preoperative management, and the predictive value of the lymph node metastasis rate in patients with pancreatic resection has been supported by studies. New scores may contribute to the postoperative management and selection of individualized therapy, which may influence clinical decisions, including neoadjuvant chemotherapy, surgical treatment, and postoperative adjuvant chemotherapy. On the one hand, the authors believe that the application of new scores in clinical work will greatly improve the accuracy of preoperative risk stratification for these patients to a certain extent. On the other hand, the addition of new scores seems to add new options for clinical work on the premise of following the guidelines and combining patients' physical status, family conditions, and their own wishes. Neoadjuvant and postoperative adjuvant therapies and treatment methods and plans are more diversified and individualized. This can prolong patients' lives, improve their quality of life, and reduce the social burden on their families.

## 6 CONCLUSION

Liver metastasis score may be an independent predictor of postoperative liver metastasis in patients with pancreatic head cancer. Survival and LNR scores may be independent predictors of long‐term postoperative survival in patients with pancreatic head cancer, with the LNR score appearing to have better long‐term survival than the survival score. There was no significant correlation between survival and LNR scores and TNM stage

## AUTHOR CONTRIBUTIONS

Dan Jiang performed the experiments, prepared the figures and tables, and drafted the manuscript. Xiaona Fan, Hengzhen Li, Jinshuang Liu, and Wenjing Zhang revised the manuscript. Pengfei Li, Yang Zhou, and Kaige Chen obtained images and conducted measurements. Yisheng Dai and Ning Sun collected clinical data. Zhiwei Li designed the experiments and supervised the project. All authors have read and approved the final version of this manuscript.

## FUNDING INFORMATION

This work was supported by the Wu Jieping Medical Foundation (320.6750.2020‐12‐6) and Beijing Medical Award Foundation (YXJL‐2020‐0785‐0185).

## CONFLICT OF INTEREST

The authors declare no conflicts of interest.

## ETHICS APPROVAL

This study was approved by the Ethics Review Board of Harbin Medical University (ethics number: KY2021‐24). All patients signed an informed consent form for the second use of medical history data/biospecimens in the process of diagnosis and treatment and agreed to provide diagnosis and treatment information for scientific study. The requirement for exemptions from informed consent for this study was waived owing to the retrospective design of this study. The privacy and identity information of the patients are protected.

## Data Availability

The data that support the findings of this study are available upon request from the corresponding author. The data are not publicly available due to privacy or ethical restrictions.

## Appendix

**TABLE A1:** Univariate analysis of characteristics and liver metastasis score of patients with pancreatic head cancer

Factor	Hepatic metastasis score			*p*‐value
	0 (*n* = 36)	1 (*n* = 46)	2 (*n* = 45)	
AST	69.5^a^	70^a^	73^a^	0.068
ALT	121.4^a^	118.9^a^	124^a^	0.079
ALB	38^a^	38^a^	38.3^a^	0.110
ALP	286.5^a^	283^a^	316^a^	0.053
LDH	177^a^	177^a^	180^a^	0.023
γ‐GGT	357.1^a^	356.5^a^	387^a^	0.068
PT	11.75^a^	11.8^a^	11.9^a^	0.017
APTT	28.05^a^	28^a^	27.5^a^	0.044
TT	17.5^a^	17.5^a^	17.4^a^	0.199
INR	1.02^a^	1.02^a^	1.03^a^	0.010
TBIL	93.31^a^	97.7^a^	85.48^a^	0.072

Abbreviations: ALB, albumin; ALP, alkaline phosphatase; ALT, alanine aminotransferase; APTT, activated partial thromboplastin time; AST, aspartate aminotransferase; INR, international normalized ratio; LDH, lactate dehydrogenase; PT, prothrombin time; TBIL, total bilirubin; TT, thrombin time; γ‐GGT, gamma‐glutamyl transferase.

^a^ Median.

**TABLE A2:** Univariate analysis of characteristics and LNR score of patients with pancreatic head cancer

Factor	LNR score			*p*‐value
	0 (*n* = 17)	1 (*n* = 101)	2 (*n* = 31)	
Age (year)	59^a^	58^a^	59^a^	0.181
Sex (male/female)	13/4	56/45	21/10	0.156
BMI	3/10/4	16/55/30	6/14/11	0.875
Underlying disease (+/−)	5/12	48/53	12/19	0.304
Liver disease (+/−)	9/8	43/58	12/19	0.631
Child‐Pugh stage (A/B)	16/1	83/18	25/6	0.359
Tumor length to diameter (≤3/>3 cm)	9/8	51/45	18/10	0.053
CEA	3.145^a^	3.21^a^	3.22^a^	0.973
CA199	163.8^a^	166.8^a^	163.8^a^	0.976
Neoplasm staging (I/II/III)	7/10/0	38/59/4	12/16/3	0.773
Degree of tumor differentiation				0.301
Nerve invasion (+/−)	6/11	32/69	8/23	0.753
Postoperative hepatic metastases (+/−)	5/12	26/75	4/27	0.355
Postoperative distant metastases (+/−)	7/10	48/53	8/23	0.091
PLR	143.9^a^	143.9^a^	143.9^a^	0.971
NLR	2.77^a^	2.77^a^	2.72^a^	0.976
HGB	126.45^a^	125^a^	125^a^	0.755

Abbreviations: BMI, body mass index; CA 19‐9, carbohydrate antigen199; CEA, carcinoembryonic antigen; HGB, hemoglobin; LNR, lymph node ratio; NLR, the peripheral blood neutrophil to lymphocyte ratio; PLR, the preoperative platelet to lymphocyte ratio.

^a^ Median.

**TABLE A3:** Univariate and multivariate analysis of postoperative liver metastasis

Factor	Univariate			Multivariate		
	95% CI	OR	*p*‐value	95% CI	OR	*p*‐value
AST	0.996–1.004	1.000	0.915			
ALT	0.998–1.003	1.000	0.883			
ALB	0.909–1.076	0.989	0.800			
ALP	0.997–1.001	0.999	0.154			
LDH	0.989–1.007	0.998	0.613			
γ‐GGT	0.999–1.001	1.000	0.991			
PT	0.668–1.363	0.954	0.796			
APTT	0.940–1.154	1.042	0.435			
TT	0.667–1.188	0.890	0.430			
INR	0.007–40.546	0.544	0.782			
TBIL	0.996–1.004	1.000	0.965			

Abbreviations: ALB, albumin; ALP, alkaline phosphatase; ALT, alanine aminotransferase; APTT, activated partial thromboplastin time; AST, aspartate aminotransferase; INR, international normalized ratio; LDH, lactate dehydrogenase; PT, prothrombin time; TBIL, total bilirubin; TT, thrombin time; γ‐GGT, gamma‐glutamyl transferase.
